# Real clinical experience of inhaled esketamine in depression and chronic pain. A retrospective study

**DOI:** 10.1192/j.eurpsy.2025.1303

**Published:** 2025-08-26

**Authors:** S. Benavente López, I. Pedrero Torrejon, E. Toro Carrasco, A. Lara Fernández, A. Parra González, M. Mejia Quiterio, A. Viñas Arboleda, E. Baca García

**Affiliations:** 1 Department of Psychiatry, Hospital Universitario Infanta Elena, Madrid, Spain

## Abstract

**Introduction:**

Chronic pain is very prevalent in the population, with an estimated prevalence of 20.5% of the adult population experiencing pain every day or most days. Chronic pain is one of the main factors that generate dysfunctionality in patients, and it can even lead to dependence in activities of daily living. Chronic pain is strongly associated with depression, and can induce a major depression which, due to its characteristics, will become chronic. Several studies have shown that intravenous ketamine can be effective in the treatment of chronic pain. Major depression associated with chronic pain has a worse outcome and is more frequently associated with resistance to pharmacological treatment, making the management of these patients a clinical challenge.

The present study shows the response in a series of cases with major depression and chronic pain treated with inhaled esketamine, showing their evolution through the Montgomery-Asberg Depression Rating Scale (MADRS) and Fibromyalgia Impact Questionnaire (FIQ) as main outcomes.

**Objectives:**

The main objective of this study is to describe the use of inhaled esketamine in patients with major depression and chronic pain in real clinical practice, using the MADRS and FIQ as main outcomes to show the evolution.

**Methods:**

Retrospective descriptive study with a sample selected by non-probabilistic consecutive sampling, retrospective type, in a time interval of 2 years. The patients selected were those who completed treatment with inhaled esketamine and had a diagnosis of major depression and a diagnosis of chronic pain at Hospital Universitario Infanta Elena. A descriptive analysis was performed. Mean and standard deviation were calculated for quantitative variables and N and percentage for categorical variables.

**Results:**

A total of 5 patients with a diagnosis of major depression and chronic pain (n: 5) all treated with inhaled esketamine were included in the study. The efficacy of inhaled esketamine was high in the reduction of pain during the months of treatment with it, presenting an improvement in the MADRS and the FIQ. At the end of treatment, there was a significant difference between patients who had made changes in their daily life habits (sport, improved daily activities, improvement in the quality of life) and those who had not made changes in their daily life habits.

**Conclusions:**

This study shows that inhaled esketamine may be a useful drug in the treatment of major depression associated with chronic pain. Treatment with esketamine improved depressive symptoms and also chronic pain. A significant influence of changes in lifestyle habits was observed in maintaining the response to pain relief once treatment with esketamine was finished. Longitudinal studies must be carried out to confirm this hypothesis.

**Disclosure of Interest:**

None Declared

